# Cognitive Decline in Relation to Psychological Wellbeing and HIV Disease- and Treatment Characteristics in HIV-Infected Patients on cART: A One-Year Follow-Up Study

**DOI:** 10.1007/s10461-016-1583-7

**Published:** 2016-10-17

**Authors:** Marloes A. M. Janssen, Peter P. Koopmans, Roy P. C. Kessels

**Affiliations:** 1 Department of Medical Psychology 840, Radboud University Nijmegen Medical Center, P.O. box 9101, 6500 HB Nijmegen, The Netherlands; 2 Department of Internal Medicine, Radboud University Medical Center, Nijmegen, The Netherlands; 3 Donders Institute for Brain, Cognition and Behaviour, Radboud University, Nijmegen, The Netherlands

**Keywords:** Cognitive decline, Follow-up, Wellbeing, HIV, RCI

## Abstract

The objectives of the current study were to examine cognitive decline in relation to psychological wellbeing, HIV disease and treatment characteristics and baseline variables over a one-year period of time in a group of HIV-infected patients on long term cART with undetectable viral load in comparison to a HIV-negative control group. Eighty-two of 95 patients and 43 of 55 controls who completed a baseline assessment for the Art-NeCo study underwent a follow-up neuropsychological assessment. A repeated-measure general linear model analysis was performed to compare the performance at follow-up in comparison to baseline between the patients and controls. Reliable change indices were computed as a measure of significant change in cognitive function. Compared to controls, patients overall performed worse on the domain speed of information processing. In the patient group a worse performance at follow-up was present for the verbal fluency domain compared to the controls, in the absence of a baseline group difference. For the executive function domain, no group differences were found at follow-up, but the patients performed worse than the controls at baseline. We found that cognitive decline was related to more frequent use of recreational drugs and a somewhat heightened level of irritability and more somatic complaints at baseline. However, the decliners did not differ from the non-decliners on any of the HIV-related variables.

## Introduction

HIV-associated neurocognitive disorders (HAND) are still frequently found in the cART era [1, 2]. Whereas the prevalence of most severe forms of HAND have vastly declined, milder forms are still being reported. Over the years, deficits in various cognitive domains have been demonstrated, with speed of information processing, attention, memory and executive function showing the strongest impairments [2–5].

Several longitudinal studies on neurocognitive decline and potential predicting factors have been performed [4, 6, 7]. In their review Cysique et al. [8] found that cART is associated with stable neuropsychological functioning over time, especially in clinically stable patients with undetectable plasma viral loads over a long period of time. Nevertheless, a longitudinal study with a large study sample showed cognitive decline at follow-up assessments in 21 % of patients who were cognitively unimpaired at baseline [7]. A study in HIV-infected patients with HIV-related cognitive impairment showed persistent impairment in 62.8 % of the patients at follow-up (6–124 months) despite treatment with cART [4].

Some studies used available normative data to determine significant and clinically relevant cognitive change in individual patients. In one study, 192 patients with HIV and 101 HIV negative controls from China were followed for 1 year and the results on cognitive assessment of the controls were used to derive regression-based norms for change [9]. The scores that were obtained using this approach were adjusted for practice effects, regression toward the mean, and other factors that may have an effect on normal test–retest variability in neurologically stable people (e.g. test–retest interval, demographics, and overall baseline neuropsychological competence). Twenty-seven percent of the patient group showed significant cognitive decline over a one-year period. This decline was predicted by having AIDS at baseline, lower nadir CD4 cell count and failure of viral suppression on cART. Cognitive decline was also related to decreased independence in activities of daily living. Another study using a subset of patients from the CHARTER cohort also investigated cognitive change using a similar approach. Cognitive change of 436 HIV-infected patients who underwent 4 to 7 study visits was determined with z-scores based on published normative data [10]. Results showed cognitive decline in 22.7 % of the patients, 60.8 % remained stable and 16.5 % improved. Predictors for cognitive change were time-dependent treatment status (ART status, immunosuppression, and plasma and CSF HIV RNA load), indicators of disease severity (current hematocrit, albumin, total protein and aspartate aminotransferase), premorbid IQ, depressive symptoms, and lifetime psychiatric diagnoses.

So far, previous studies that used a statistical approach to determine cognitive change over time included mixed cohorts consisting of both patients on cART and cART naïve patients. In the current study cognitive change was investigated in a group of HIV-infected patients on long term cART treatment with undetectable viral load in comparison to a matched HIV-negative control group. The aim of this study is twofold. First, we examined cognitive decline over a one-year period by calculating test–retest scores for a comprehensive neuropsychological assessment. Second, we identified which HIV disease and treatment characteristics as well as which psychological and baseline variables are associated with this decline.

## Methods

### Participants

All participants were recruited as part of the Art-NeCo study (a prospective Dutch cohort study on *Ne*uro*Co*gnition in HIV-infected patients on long term effective combination antiretroviral therapy; c*ART*) [5]. Baseline assessment was performed in 95 HIV-infected patients and 55 HIV-negative controls. An extensive description of all inclusion and exclusion criteria have been published previously [5]. Of these participants, 82 patients and 43 controls underwent a follow-up neuropsychological assessment yielding a 16, 7 % (25/150) attrition rate. Reasons for dropping out were lack of time, lack of interest to participate again and personal reasons like problems at work or at home, unrelated to HIV status or symptom validity testing. No significant differences were found between completers and non-completers on the baseline variables age, education level, sex distribution, sexual orientation, performance on cognitive domains at baseline, nadir CD4, time on cART or duration of HIV infection (p > .05). At baseline, 34 patients fulfilled the criteria for asymptomatic neurocognitive impairment and five for mild neurocognitive disorder. None of the patients fulfilled the criteria for HIV-associated dementia. The cognitive decrements were mild in nature and cognitive domains that are typically affected by HIV infection like executive functioning and memory were unimpaired in our patient sample [5].

### Procedures

At both study visits participants completed a comprehensive neuropsychological assessment measuring eight cognitive domains (speed of information processing, learning, memory, executive function, attention/working memory, motor function, visuoconstruction and verbal fluency). Also, a self-report questionnaire was completed, the symptom checklist—revised (SCL-90-R), to measure psychological distress and somatic complaints. At baseline, a questionnaire on alcohol and other recreational drug use was completed by all participants and a rapid HIV test was performed in all controls. Also, participants without contraindications underwent an MR examination at baseline and symptom validity testing was performed. Baseline details of the Art-NeCo in- and exclusion criteria, the neuropsychological test battery and the MRI analyses have been reported in a previous publication [5]. Medical ethical approval for this study and written informed consent from all study participants were obtained (CMO Region Arnhem–Nijmegen #2011/267).

### Statistical Analyses

Raw test scores of the individual tests of the baseline and follow-up neuropsychological test battery were transformed into standardized z-scores. Test scores were transformed on the basis of the baseline test score distribution of the patient- and control group taken together, with higher scores indicating a better performance for all tests. Cognitive domain scores were computed by averaging the contributing tests to each of the cognitive domains [11].

A repeated measures General Linear Model (GLM) was executed to compare the performance at follow-up in comparison to baseline between the patients and controls. Age, sex and estimated premorbid IQ were entered as covariates. Alpha was set at 0.05 for all analyses.

### Determination of Cognitive Decline

Next, analyses were performed to establish which patients deteriorated at follow-up compared to baseline, using the change in performance in the controls as benchmark. Reliable change indices (RCIs) were computed per patient as a measure of individual significant change on the cognitive tests after a one-year retest interval. According to the procedure outlined in Chelune et al. [12], difference scores of the individual test scores between the first (*T1*) and second (*T2*] assessment were calculated for each individual patient (ptx) and a mean difference score was calculated for the control group as a whole (ct). The difference score for each patient was subtracted by the mean difference score of the control group as whole, using the following formula: (*T2*_ptx_−*T1*_ptx_) – (*T2*_ct_−*T1*_ct_). Next, the standard error of difference (*Sdiff*) was calculated for each of the test scores per patient, following the procedures outlined in Maassen [13], using the following formula: *Sdiff* = $\sqrt {\left( {SD T1_{ct}^{2} } \right) + \left( {SD T2_{ct}^{2} } \right) \times (1 - rT1 \cdot T2)}$

Finally, the difference score of each patient per test was divided by the standard error of difference in order to calculate the RCI index per test per patient. Next, individual RCIs per test were averaged into the eight cognitive domains. Patients with a mean RCI domain score ≥ −1.645 [12] were considered as ‘cognitive decliners’ on that domain. Subsequently, individual patients were classified as either overall cognitive decliner, defined as showing a reliable decline on at least one cognitive domain, or non-decliner. Subsequently, overall cognitive decliners were compared to non-decliners on the SCL-90-R subscale performance, alcohol and drug use, HIV disease and treatment variables, age and MRI measures at baseline.

## Results

Only participants who passed symptom validity testing were included in all analyses [5]. Mean follow-up interval was 12.2 months (range 10.1–18.6 months). Table 1 shows the baseline demographic characteristics, SCL-90-R scores and MRI measures of the patients and controls who participated in the follow-up assessment and the HIV disease and treatment characteristics for the patients.

**Table 1:** Demographic variables, SCL-90-R, MRI correlates and treatment characteristics at baseline

Characteristic	Patients (*n* = 82)	Controls (*n* = 43)
Age (years) [mean (SD)]	48.7 (9.9)	51.4 (11.1)
Sex		
Men	72 (87.8 %)	34 (79.1 %)
Women	10 (12.2 %)	9 (20.9 %)
Education level [median (range)]^3^	6 (2–7)*	6 (4–7)
Estimated IQ [mean (SD)]	98.7* (14)	106.8 (11.4)
Nadir CD4 cell count (cells/µL) [mean (IQR)]	222 (100–310)	–
Duration HIV-infection (years) [mean (SD)]	9.6 (6.4)	–
Duration cART treatment (years) [mean (SD)]	8.1 (5.8)	–
Regular alcohol use^1^	25 (30.5 %)	18 (41.9 %)
Regular drug use^2^	7 (8.5 %)	3 (7 %)
SCL-90-R [mean (SD)]		
Total	131 (38.3)	114 (26.2)
Agoraphobia	8 (2.3)	8 (1.2)
Anxiety	14 (4.4)	12 (3.3)
Depression	25 (8.9)	21 (6.6)
Somatic complaints	18 (5.5)	15 (4.3)
Cognitive performance difficulty	14 (5.1)	12 (3.4)
Interpersonal sensitivity	25 (9.1)	23 (6.4)
Anger-hostility	8 (2.3)	7 (1.5)
Sleep disturbances	6 (2.6)	4 (1.6)
MRI volumetry [mean (SD)]		
Grey matter volume^4^	619.3 (46.1)	613.8 (39.6)
White matter volume^4^	602.8 (39.8)	610.5 (25.7)

^1^Regular alcohol use is use of alcohol for three or more times a week or binge drinking on two subsequent days

^2^Regular drug use is use of a drug for four times or more times a month

^3^Education level was recorded using seven categories based on the Dutch educational system which uses education levels instead of years of formal education (approximate Anglo–Saxon equivalent in years of education is presented between brackets): Level 1 = less than 6 years of primary education (1–5); Level 2 = completed primary education (6), Level 3 = more than 6 years of primary education, without a secondary school diploma (7–8), Level 4 = lower vocational training (7–9); Level 5 = advanced vocational training or lower professional education (7–10); Level 6 = advanced professional training or upper secondary school (7–17); Level 7 = academic degree (>18 years)

^4^Relative Grey- and White matter: grey matter volume/total intracranial volume (TIV) × all patient TIV mean, white matter volume/total intracranial volume (TIV) × all patient TIV mean

* *p* < 0.05

### Baseline Versus Follow-up Cognitive Performance

**Fig. 1:**
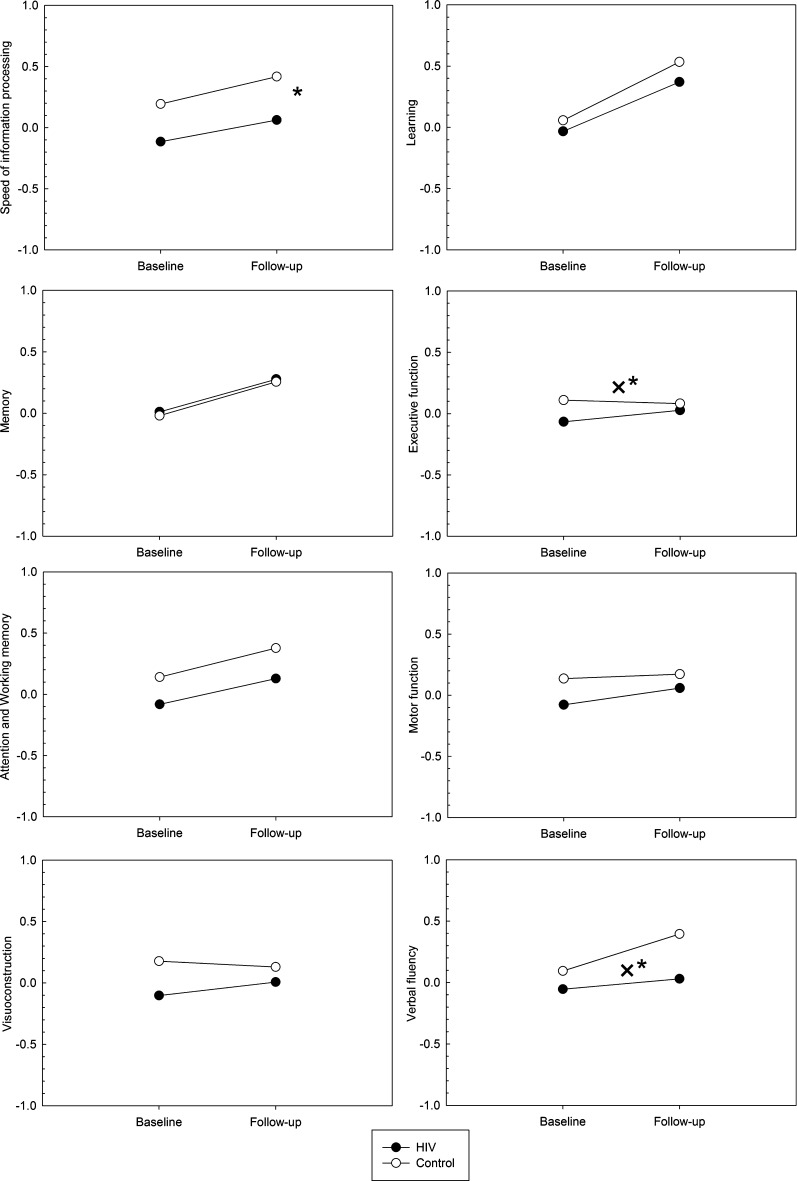
Mean cognitive domain scores for the patients and controls at baseline and one-year follow-up (* = significant main effect of group, p < 0.05; ×* = significant group × time interaction, p < 0.05)

Figure 1 shows the baseline and follow-up cognitive performance for the patients and controls for the individual cognitive domains. Repeated-measure GLM analyses showed an overall better performance at follow-up than at baseline for both groups for the domains speed of information processing, executive function and visuoconstruction (F-values > 4.6, p < .05). An overall group difference was found on the domain speed of information processing (F = 5.1, p = .025, η_p_^2^ = .041), with the patients performing worse than the controls. A significant group × time interaction was found for the domains executive function (F = 10.1, p = .002, η_p_^2^ = .077) and verbal fluency (F = 4.2, p = .04, η_p_^2^ = .034). Post-hoc t-tests demonstrated that at follow-up, but not at baseline, the performance of the patients on the verbal fluency domain was worse than the controls (t (123) = −2.129, p < .05 and t (123) = −0.666, n.s., respectively). Between-group differences were found at baseline, but not at follow-up, for the domain executive function (t (123) = −2.249, p < .05 and t (123) = −0.493, n.s., respectively).

### Cognitive Decliners Versus Non-decliners

Of the total group of 82 patients, 7 patients showed cognitive decline on the domain verbal fluency (8.5 %), 2 on the domain visuoconstruction (2.4), 1 on the domain speed of information processing (1.2 %) and 1 on motor function (1.2 %). No decliners were found in any of the other domains. In total, 11 patients were classified as being overall cognitive decliners (13.4 %). These cognitive decliners were subsequently compared to the non-decliners on SCL-90-R subscale performance, alcohol and drug use, HIV disease and treatment variables, age and MRI measures at baseline. The cognitive decliners reported more complaints on the subscales somatic complaints (F(1,81) = 5.4, p < .05) and anger-hostility (F(1,81) = 5.9, p < 0.02) of the SCL-90-R at baseline compared to the non-decliners. With respect to any of the other baseline characteristics, the proportion of frequent users of recreational drugs was higher in the cognitive decliners than the non-decliner group (χ^2^(1) = 6.56, p < .01). No differences were found between both groups on any of the other baseline or HIV-related baseline characteristics.

## Discussion

The present study investigated cognitive change over a one-year period in a virologically stable HIV-infected patient group in comparison to a healthy matched control group. Also, we examined whether HIV disease and treatment characteristics and psychological baseline variables were associated with cognitive decline.

Compared to the controls, the patients overall performed worse on the domain speed of information processing, but no group × time interaction was found for this domain. For the domain verbal fluency, a group × time interaction effect was found, showing a worse performance at follow-up in the patient group compared to the controls, in the absence of a baseline group difference. For the domain executive function, no group difference was found at follow-up, but the patients performed worse than the controls at baseline. Our findings clearly show that a lower performance in speed of information processing, both at baseline and follow-up, is a robust finding, in line with previous studies [2, 14] that also report impairments in this domain in HIV-infected individuals on cART. With respect to the follow-up performance difference in the domain verbal fluency for the patients, it is interesting to note that while both groups improve on an absolute level, the improvement in the patients was smaller than in the controls. Also, it should be noted that this domain only consists of one test, which also relies on processing speed. In the present subsample of the Art-NeCo sample, a baseline difference between patients and controls was found for executive function, a finding that disappeared at follow-up. Interestingly, this baseline difference was not found in the total Art-NeCo group [5], indicating that this effect may not be very reliable.

At an individual level, most cognitive decliners (based on RCI analyses using the control group performance as reference) were found in the domain verbal fluency. Comparing the patients who reliably declined on at least one of the cognitive domains with those who remained cognitively stable, we found that the decliners reported more psychological complaints, specifically related to irritability towards others and somatic complaints at baseline. However, the decliners did not differ from the non-decliners on any of the HIV-related variables, such as nadir CD-4 cell count, duration of infection and years of cART use. This suggests that the higher level of somatic complaints cannot be explained by illness-related somatic factors. The decliners, however, were more often frequent users of recreational drugs than the non-decliners, which may potentially underlie the somewhat lower somatic wellbeing in this subsample [15]. However, it should be stressed that the total group of recreational drug users and the number of overall cognitive decliners were small, therefore this result should be interpreted with caution. No differences between decliners and non-decliners were found on depressive symptoms and anxiety, two psychological domains that are frequently related to worse cognitive performance [16–18].

A limitation of the current study is the relatively short interval of one year between the baseline and follow-up assessment. Particularly in a well-treated HIV-infected patient sample without co-morbidities, longer follow-up periods of five or even ten years may contribute to our knowledge of long-term consequences of HIV infection on cognitive functions in patients who are stable on cART. On the other hand, Becker et al. [19] demonstrated cognitive decline already after a six-month follow-up assessment, a finding that was found to be related to medication adherence. This further emphasizes the need and clinical relevance to examine cognitive decline over time in HIV-infected patients.

In conclusion, the findings of the current study show that cognitive decline at group level is small. We only found a decline in speed of information processing after one year. At an individual level, cognitive decline was present in only a small number of patients and was related to somewhat more psychological distress and more frequent use of recreational drugs. No relation between cognitive decline over time and HIV disease and treatment characteristics was found.
